# Supplement to the Veterinary Medical Register

**Published:** 1882-01

**Authors:** 


### SUPPLEMENT

TO THE

### VETERINARY MEDICAL REGISTER

—OF THE—

### UNITED STATES.

COMPILED FOR THE

*JOURNAL OF COMPARATIVE MEDICINE.*

[COPYRIGHTED: ALL RIGHTS RESERVED.]

Arrowsmith, William H., Jersey City, N. J.
Ash, Thomas J., D.V.S., Brooklyn, E. D., N. Y.
Bartlett, E., Springfield, Mass.
Bates, G. W., V.S., Wellington, Mo.
Birch, W. A., D.V.S., Philadelphia, Pa.
Boardman, W., V.S., Blackburry Station, Ill.
Bolton, A. H., V.S., Mankato, Minn.
Boone, E. B., Baltimore, Md.
Brackett, E. J., V. S., Lewiston, Maine.
Brackin, J. A., V.S., Pittsfield, Mass.
Bray, T. A., D.V.S., Morris, Ill.
Brooks, Frank E., V.S., Rochester, N. Y.
Burns, C., W. H. F. Co., Baltimore, Md.
Byrne, C., M.R.C.V.S., Boston, Mass.
Chase, T. P., V.S., Rock Island, Ill.
Cladfelter, R., D.V.S., Philadelphia, Pa.
Cleaver, K. H., V.S., Allentown, Pa.
Conklin, W. A., D.V.S., Ph.D., New York City, N. Y.
Cook, Andrew S., V.S., Binghamton, N. Y.
Coppis, G. W.,V.S., Madisonburg, O.
Corbyn, C. H., Philadelphia, Pa.
Counselman, W. H., Baltimore, Md.
De Vore, David L., V. S., Red Oak, Ohio.
Doan, Henry C., V.S., Iowa.
Farrell, H. Y., Savannah, Ga.
Fay, James, Philadelphia, Pa.
Felton, Charles, Philadelphia, Pa.
Foelker, Samuel, V.S., Allentown, Pa.
Folsetter, William, V.S., Evansville, Ind.
Forgason, T. C., V.S., Auburn, N.Y.
French, O. B., V.S., Bloomfield, N.Y.
Galbraith, Arthur D., V.S., Ewington, Ind.
Gould, T., St. Paul, Minn.
Gowland, George, V.S., Seneca Falls, N. Y.
Green, Chas. S., V.S., Richmond, Ill.
Greene, E., V.S., Bloomingvalley, Pa.

Guiteau, W., V.S., Battlecreek, Mich.
Gunn, D. A., Bowling Green, Ohio.
[illegible], A., V.S., Louisville, Ky.
Hayga[illegible]d, E. T., V.S., Lexington, Ky.
Haygard. J. R., V.S., Lexington, Ky.
Hacker[illegible]ger, H., V.S., Catasauga, [illegible]
H[illegible]erson, Matthew J., V.S., Syracuse, N. Y.
Herbert, S. R., V.S., Cohoes, N. Y.
Hood, T. A., V.S., Ogdensburgh, N. Y.
Hoskins, W. H., D.V.S.
Howard, R. D., V.S., Castile, N. Y.
Humphries, J., V.S., Lockhaven, Pa.
Hutchings, Robert C., V.S., Watertown, N. Y.
Jennings, R., Detroit, Mich.
Jennings, R., Jr., M.D., V.S., Pittsburgh, Pa.
Jordan, Martin, V.S., U. S. Army.
Judson, J. H., M.D., V.S., Polo, Ill.
King, B. F., V.S., Little Silver, N. J.
Langtry, W., V. S., Fort Wayne, Ind.
Lee, William, M.D., V.S., Philadelphia, Pa.
Lewis, Richard, Vicksburg, Kalnago County, Mich.
Livesay, M. C., Eureka, Mich.
Loomis, E., V.S., Elmira, N. Y.
Levette, H., Philadelphia, Pa.
McKenny, Richard, V.S., Michigan.
McLeod, Alexander, V.S., Jackson, Mich.
McNally, M., V. S., Houston, Texas.
McIntosh, James, D. V. S., Jersey City, N. J.
MacWhinney, James, V.S., Buffalo, N. Y.
Maguire, A. B., V.S., Joliet, Ill.
Mann, Otis, V.S., Springfield, Mass.
Martin, James, V.S., Lockport, N. Y.
Miller, Joseph, V.S., Seville, Ohio.
Moor, A., V.S., Mansfield, Ohio.
Morrison, T. A., V.S., LaSalle, N. Y.
Morse, — —, Providence, R. I.
Osgood, F. H., Springfield, Mass.
Phillips, J. B., V.S., 565 North Twenty-fifth st., Philadelphia, Pa.
Pierce, B. A., V.S., Creston, Ill.
Prentice, E., V.S., Chicago, Ill.
Ramsey, J., Boston, Mass.
Raynor, James B., D.V.S., Westchester, Pa.
Regen, Francis, M.D., V.S., Washington, D. C.
Robson, G. L., V.S., Penn Yann, N. Y.
Rogers, E. S., V.S., Staynor, Ont.
Rowe, N., M.D., V.S., Chicago, Ill.
Salmon, D. E., D.M.V., Asheville, N. C.
Savale, E. N., D.V.S., Morristown, N. J.
Schaufler, Charles, Philadelphia. Pa.
Simmons, T. W., M.D., M.R.C.V.S., Boston, Mass.
Simon, Th., D.V.S., New York City, N. Y.
Smead, C. D., V.S., Logan, N. Y.
Smeall, A. N., V.S., Seville, Ohio.
Smithers, P., V.S., St. Louis, Mo.
Sommerville, Robert, V.S., Buffalo, N. Y.
Stevens, Elwood, Philadelphia, Pa.
Sutterby, H., V.S., Batavia, N. Y.
Thomas, W. A., B.V.M., Lincoln, Neb.
Thompson, Warwick M., V.S., Denver, Col.
Van Buskirk, F., V.S., Allentown, Pa.
Voltz, E. H., Baltimore, Md.
Washburn, N. R., Springfield, Mass.
Watson, J., Philadelphia, Pa.
Welsh, John, V.S., Ohio.
Whellpley, Samuel, V.S., New York City, N. Y.
White, M., Bowling Green, Ohio.
Whitehead, Robert W., V.S.. Ohio.
Whitney, F. E., Springfield, Mass.
Whitney, William W., Springfield, Mass.

